# Novel compound shows in vivo anthelmintic activity in gerbils and sheep infected by *Haemonchus contortus*

**DOI:** 10.1038/s41598-022-17112-3

**Published:** 2022-07-29

**Authors:** Elora Valderas-García, Nerea Escala, María Álvarez-Bardón, Verónica Castilla-Gómez de Agüero, Maria Cambra-Pellejà, Laura González del Palacio, Raquel Vallejo García, Jennifer de la Vega, Arturo San Feliciano, Esther del Olmo, María Martínez-Valladares, Rafael Balaña-Fouce

**Affiliations:** 1 Instituto de Ganadería de Montaña, CSIC-Universidad de León, 24346 Grulleros, León, Spain; 2 Departamento de Ciencias Biomédicas, Facultad de Veterinaria, Universidad de León, 24071 León, Spain; 3 Departamento de Ciencias Farmacéuticas: Química Farmacéutica, Facultad de Farmacia, Universidad de Salamanca, CIETUS, IBSAL, 37007 Salamanca, Spain; 4 Departamento de Sanidad Animal, Facultad de Veterinaria, Universidad de León, 24071 León, Spain

**Keywords:** Drug screening, Parasitic infection

## Abstract

The control of gastrointestinal nematodes in livestock is becoming increasingly difficult due to the limited number of available drugs and the rapid development of anthelmintic resistance. Therefore, it is imperative to develop new anthelmintics that are effective against nematodes. Under this context, we tested the potential toxicity of three compounds in mice and their potential anthelmintic efficacy in Mongolian gerbils infected with *Haemonchus contortus*. The compounds were selected from previous in vitro experiments: two diamine (AAD-1 and AAD-2) and one benzimidazole (2aBZ) derivatives. 2aBZ was also selected to test its efficacy in sheep. In Mongolian gerbils, the benzimidazole reduced the percentage of pre-adults present in the stomach of gerbils by 95% at a dose of 200 mg/kg. In sheep, there was a 99% reduction in the number of eggs shed in faeces after 7 days at a dose of 120 mg/kg and a 95% reduction in the number of worm adults present in the abomasum. In conclusion, 2aBZ could be considered a promising candidate for the treatment of helminth infections in small ruminants.

## Introduction

Gastrointestinal parasitism, especially caused by helminth species, is a major constraint in livestock production systems. These infections impair animal health seriously causing reductions in weight, meat and milk production and fertility^1^. One of the most important species, not only because of its global distribution but also because of its pathogenic potential, is *Haemonchus contortus*^2^. This abomasum parasite has a blood-feeding behavior that causes anemia and can lead to death in heavily infected animals^1,3^. In Europe, annual economic losses produced by helminth infections can reach 357 million euros in sheep and 1.8 billion euros in livestock in general^4^. Globally, these losses have been estimated at approximately 10 billion euros per year^5^. Currently, anthelmintics applied in the veterinary field basically include benzimidazoles, imidazothiazoles and macrocyclic lactones^6^. Their good therapeutic profiles including broad-spectrum of action, good tolerability and low costs are in part responsible for their excessive use for a long time. This has led to the development of varying degrees of drug resistance among nematodes for all groups of anthelmintics compromising their efficacy and control^7–11^. Therefore, the introduction of new drugs is urgently required to overcome the resistance to the current marketed drugs. However, despite this urgent need for innovation, their development is very slow since only three new compounds have been approved as anthelmintics in the last 20 years: emodepside^12^, monepantel^13^ and derquantel^14^. One of the main reasons is that drug discovery requires large sources of investment and funding^15^.

The objective of the current experiment was to assess the tolerance and the in vivo anthelmintic potential of three novel synthetic compounds identified in a previous phenotypic platform against the gastrointestinal nematode infecting sheep *Teladorsagia circumcincta*. One benzimidazole and two diamine derivatives were selected from a total of 220 compounds based on their in vitro ovicide and larvicide activity but also their high selective indexes on intestine mammalian cells^16^. Under this context, acute toxicity assays were carried out on mice while in vivo efficacy of compounds was assessed on both gerbils and sheep experimentally infected with *H. contortus*.

## Results

### Tolerance assay in mice

When the compounds were administered at a dose of 250 mg/kg body weight by using DMSO 10% and PEG300 as a drug vehicle, no mortality was observed in any of the animals, nor in the control group. Animal body weights increased progressively throughout the study period as showed in the Supplementary Table S1.

Behavioral observation of the animals did not show abnormalities during the course of the experiment in any group. No macroscopic or microscopic lesions were found on examination of isolated vital organs such as heart, liver, kidney, lungs and spleen from treated animals. Organ to body weight index was calculated and summarized in Supplementary Table S2. Statistical analysis showed that there was no significant difference (*p* > 0.05) among the groups (Tukey’s multiple comparison test).

### In vivo* bioassays on Mongolian gerbils*

As shown in Table 1, only AAD-1 had some effect in terms of L_4_ reduction since it reached a value of 16.5%. However, when focusing on pre-adult counts, 2aBZ treatment was the most effective, with a 95.63% reduction at 200 mg/kg. When the dose was reduced to 10 mg/kg the efficacy against pre-adults dropped significantly to 30.64%. No significant reduction in the number of L_4_ present in the stomach was observed for any of the compounds compared to the untreated group (*p* > 0.05) (Kruskal Wallis test). Only the groups treated with 2aBZ at a concentration of 200 mg/kg showed significant reduction in the number of pre-adults present in the stomach compared to the untreated group (*p* = 0.034) (Kruskal Wallis test).

**Table 1:** Anthelmintic effect of at a single oral administration of 2aBZ, AAD-1 and AAD-2 to gerbils infected with *H. contortus*.

Treatment type	Dose (mg/kg)	Mean worm count		% reduction	
		L_4_	Pre-adults	L_4_ (%)	Pre-adults (%)
2Abz	200	383.75 ± 55.85	3.13 ± 3.13*	0.00	95.63
2aBZ	10	348.33 ± 31.40	47.50 ± 15.21	0.00	30.64
AAD-1	200	306.25 ± 33.00	56.88 ± 25.00	3.73	20.54
AAD-1	10	314.17 ± 59.46	100 ± 18.44	1.25	0.00
AAD-2	200	265.63 ± 25.28	39.38 ± 26.49	16.50	44.99
Control		318.13 ± 19.95	71.58 ± 14.77		

Mean of L_4_ and pre-adult counts of gerbils treated with 2aBZ, AAD-1 and AAD-2 and the drug vehicle (control group), and efficacy of the treatments based on percentage reduction of worm burden. Values are presented as mean ± SEM (n = 6).

*Significant differences were observed (*p* < 0.05) (Kruskal Wallis test).

**Figure 1:**
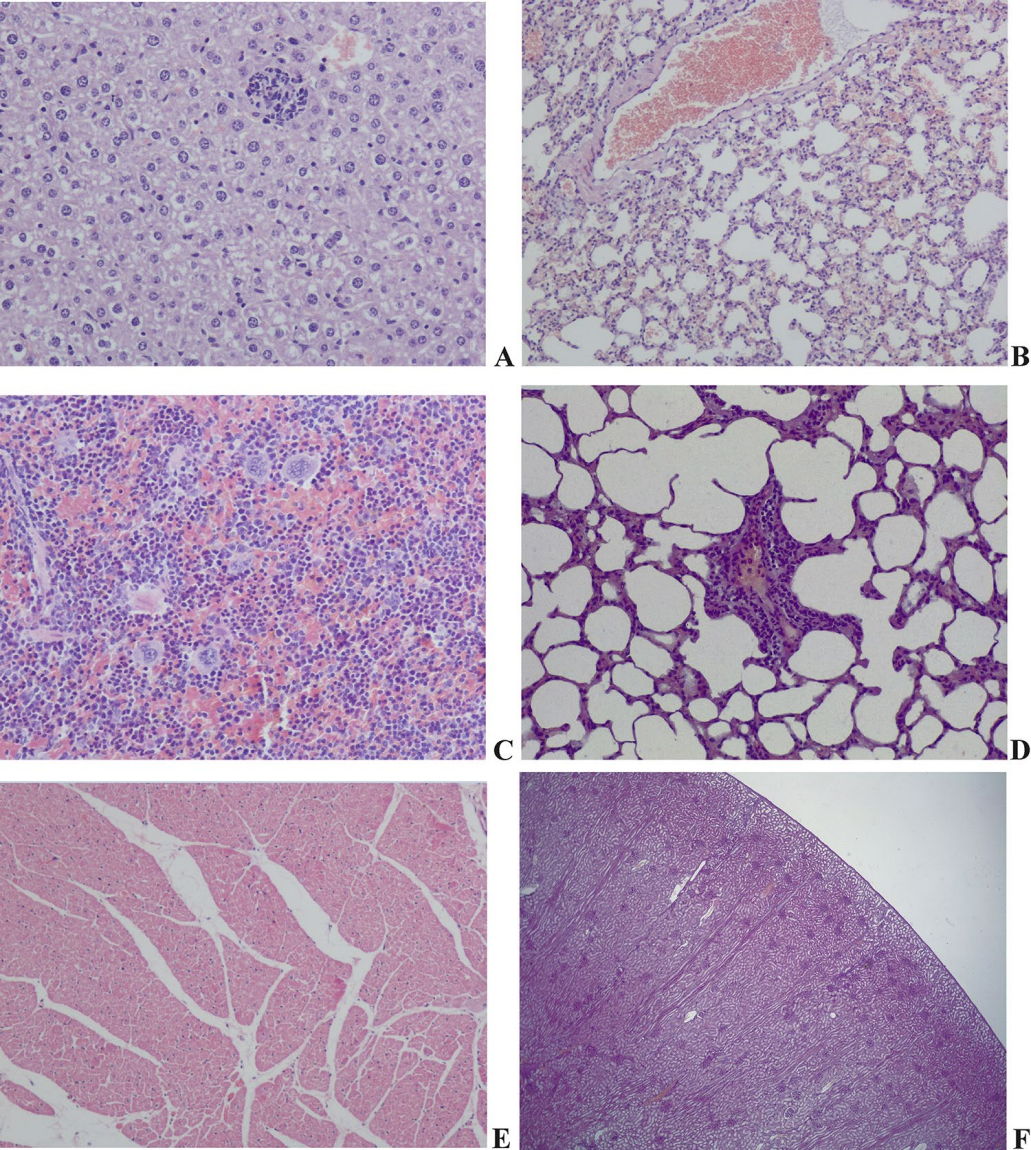
Normal histology and background findings in main organs in gerbils and sheep treated with 2aBZ and controls. No changes associated to treatment were present in any of the animals. Hematoxylin and eosin staining. **(A)** Liver of treated gerbil: normal hepatocyte structure and well ordered hepatic cords, with foci of inflammatory cells not associated with tissue damage, also present in negative controls. 100×. **(B)** Lung of control gerbil: Normal alveoli and blood vessels. Extravasated red blood cells in alveolar lumen also present in treated animals. 100×. **(C)** Spleen in control gerbil: Normal red and white pulp. Extramedullary hematopoiesis with megakaryocytes, also present in treated animals. 200×. **(D)** Lung of control sheep: Normal alveoli with low number of perivascular mononuclear cells. Also present in treated sheep. 200×. **(E)** Heart of a treated sheep: Normal cardiomyocytes and epicardium. 100×. **(F)** Kidney of a treated sheep: Normal renal structures (Glomerulus, proximal tubule and distal tubule). 40×.

The body weights of the animals were increased progressively throughout the study period as showed in Supplementary Table S3. No macroscopic lesions due to treatments were observed in the vital organs during necropsy. It was observed that compounds did not cause clinical signs of toxicity and 2aBZ did not cause microscopic lesions in the gerbil’s organs (Fig. 1).

### In vivo* bioassays on sheep*

**Figure 2:**
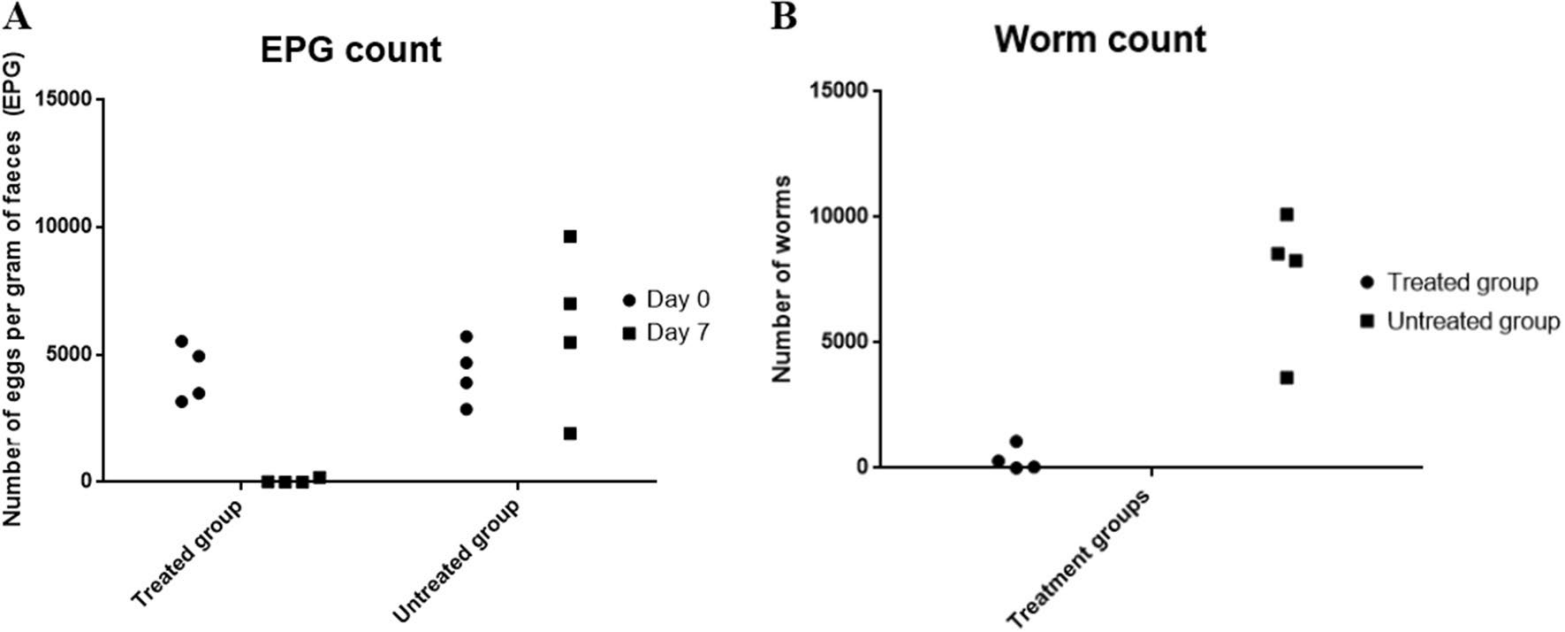
Anthelmintic effect of 2aBZ orally administered to sheep infected with *H. contortus* at a single dose of 120 mg/kg. **(A)** Number of eggs per grams of faeces (EPG count) of each animal in the treated (2aBZ at 120 mg/kg) and untreated group on day 0 and 7 post-treatment. **(B)** Number of worms recovered from each animal in the treated (2aBZ at 120 mg/kg) and untreated group on day 7 post-treatment.

FEC and worm count of each animal are shown in Fig. 2, while the means and the percentage reductions are represented in Table 2.

**Table 2:** Anthelmintic effect of 2aBZ orally administered to sheep infected with *H. contortus* at a single dose of 120 mg/kg.

Group	EPG day 0 treatment	EPG day 7th treatment	Recovered worms	% FECR	% Worm reduction
2aBZ at 120 mg/kg	4309 ± 564.7	52.5 ± 47.63*	347.5 ± 248.6*	99.13	95.45
Control (vehicle)	4316 ± 600.2	6026 ± 1604	7633 ± 1400		

The arithmetic mean EPG and worm count of sheep treated with 2aBZ were compared with untreated control. Values are presented as mean ± SEM (n = 4). EPG = egg per gram of faeces.

*FECR* Fecal egg count reduction.

*p < 0.05 significant differences were observed (Mann–Whitney U test).

The animals treated with 2aBZ averaged 52.5 *H. contortus* eggs the day 7th after treatment compared to a mean of 6026 eggs in the control group. This means a reduction of 99.13% of the number of eggs. The mean of adult worms recovered from the treated group was 347.5 compared to a mean of 7633 in the control group, which represents a worm reduction of 95.45%. Significant differences were shown between the treated and control groups regarding the number of eggs per gram of faeces (*p* = 0.029) (Mann–Whitney U test) and the number of worms at the necropsy (*p* = 0.029) (Mann–Whitney U test).

No treatment-related deaths or clinical signs of toxicity were observed after administration of the compound or during the following 7 days. No macroscopic or histological treatment related changes were observed in any of the animals studied. Low numbers of mononuclear inflammatory infiltrates in kidney, lungs and tissue around portal tracts in the liver were present in treated and negative control animals, which were considered as background lesions, together with other minimal changes (Fig. 1).

## Discussion

The occurrence of anthelmintic resistance has increased worldwide in livestock over the last decades, and further increase can be expected due to mass drug administration. Therefore, the development of new chemical entities for the treatment of these parasitic worms is urgently needed^10,17–19^. Synthesis from derivatives of known drugs with an approved use is a reasonable way to improve their properties and efficacy, as it can lead to new drugs with improved solubility and pharmacokinetics^17^. However, the risk of resistance in this case is even higher and needs to be carefully assessed^20^. Despite this, new compounds with anthelmintic activity have been developed, such as tenvermectin^21^, which belongs to the macrocyclic lactone class, or even compounds with new mechanisms of action, such as the imidazole derivative diisopropylphenyl-imidazole^22^. Progress has also been made with one of the main classes of anthelmintics currently used, the benzimidazoles. A salt of mebendazole, mebendazole nitrate, was designed to improve its water solubility^23^.

With this focus on mind, we evaluated the toxicity and anthelmintic activity of three candidates selected from previous in vitro experiments carried out in the gastrointestinal nematodes infecting sheep *T. circumcincta*. Their activity was assessed on eggs by means of the egg hatch assay and on L_3_ by means of the larval migration inhibition assay, while their cytotoxicity was tested on two different mammalian cell lines^16^. The selection criteria applied included those compounds with the highest selective indexes, meaning strongest anthelmintic potency and lowest cellular toxicity in vitro. Thus, two diamine^16^ and one benzimidazole derivatives were selected and used in the present study. Both, 2aBZ and AAD-1, showed the lowest inhibitory concentration 50 (IC_50_) in eggs with values of 1.47 and 1.01 µM, respectively, while AAD-2 reached the lowest IC_50_ in L_3_, displaying a value of 2.67 µM.

The possible toxicity of the compounds was evaluated following a similar protocol described by the “OECD Guideline for testing of Chemicals” called Acute Toxic Class method (OECD 423). The guideline advises the use of an initial dose of 300 mg/kg when no prior information on the toxicity of the compound is available. Problems with the dissolution of the compounds did not allow us to use doses higher than 250 mg/kg, especially for the benzimidazole derivative, which had to be concentrated by half and distributed in two intakes separated by half an hour. No death or changes in the behavioral pattern of the animals were found during the course of the experiment, which confirms that the Lethal Dose 50 (LD_50_) is at least, higher than 250 mg/kg. The absence of macroscopic and microscopic lesions or significant differences in organ weights also confirms the safety of the compounds at that doses as vital organs are shown to be the main target of toxic substances^24^. After confirming the safety of the compounds in mice, their potential anthelmintic activity was assessed on Mongolian gerbils and sheep.

The ultimate purpose of the study is to find a new compound active against gastrointestinal nematodes, thus the gerbil model infected with *H. contortus* was used to test the in vivo anthelmintic activity of the three compounds. This model has demonstrated to be a useful tool to study the anthelmintic efficacy and resistance of numerous commercial drugs^25–30^. In the first instance, the use of gerbils would allow us to discard inactive compounds, if any, avoiding the use of sheep in the next in vivo efficacy assay. In addition, the gerbil model requires the use of a much smaller amount of compound per animal, which is often a limiting issue and also allows testing at different doses. The election of the administered doses in the present experiment was based on a previous study carried out by Conder et al.^31^. In that study, the minimum doses effective in clearance 95% of *H. contortus* after several commercial anthelmintic treatments were established in gerbils and compared with sheep. The effective doses of marketed BZs ranged from 1.875 to 187.5 mg/kg in gerbils, so two similar doses were selected for the present study, 10 and 200 mg/kg.

Despite none of the compounds produced a significant reduction in the number of L_4_ present in the stomach of gerbils when administered at 200 mg/kg, 2aBZ caused a 95.63% reduction in numbers of pre-adults, suggesting that this compound could have effect on worm development at this larval stage or directly on more mature forms of the parasite. However, when the 2aBZ dose was reduced to 10 mg/kg, the percentage reduction decreased to 30.64%. All the compounds were well-tolerated on gerbils at the doses tested, as no clinical signs of toxicity, or macroscopic and microscopic lesions were observed.

Considering these results, the compound 2aBZ was tested in sheep at a single dose of 120 mg/kg administered orally. At day 7 post treatment, the treated group showed a reduction of 99.13% in the FEC and of 95.45% reduction in the number of worms present in the abomasum in comparison to the untreated group.

Moreover, 2aBZ was well-tolerated by sheep at this concentration as neither the macroscopic examination nor the histopathological study of the vital organs showed the presence of changes related to treatment. Moreover, according the literature, BZs present low toxicity, being the least toxic chemical family of the anthelmintic marketed drugs^32–34^.

In conclusion, 2aBZ could be a potential candidate for the treatment of *H. contortus*,—and probably other gastrointestinal nematodes infections—in ruminants, or as a starting point for the synthesis of further structurally related compounds, as it showed a significant anthelmintic activity and security at a dose of 120 mg/kg in sheep. However, numerous studies remain to be addressed, including dose-tritation trials as well as detailed pharmacological studies conducing to improve its bioavailability, which could lead to a higher in vivo efficacy at lower doses^23,35,36^. The anthelmintic efficacy also need to be investigated in a benzimidazole resistant isolate and also in other trichostrongylid species.

## Material and methods

### Animal welfare

The handling and care of the animals were conducted in compliance with Spanish Act (RD 53/2013) and European Union Legislation (2010/63/UE). The protocols were approved by the Animal Care Committee of the Universidad de León (ULE, León, Spain).

### Chemical compounds

Compounds AAD-1 and AAD-2, named AA 30 and AA 34 in a previous study in which their synthesis was described, were tested in the current study^37^ jointly with a new 2-aminobenzimidazole (2aBZ). Stock solutions of these compounds were prepared in 90% polyethylene glycol solution 300 (PEG300, Sigma-Aldrich, Spain) and 10% dimethyl sulfoxide (DMSO ≥ 99.9%, Sigma-Aldrich, Spain).

### Acute toxicity for mice

A total of 20 female Swiss albino mice (*Mus musculus*) with average weight of 25 g were caged in polypropylene boxes and given commercial rodent feed and tap water ad libitum. After an acclimatizing period of 1 week, the mice were randomly divided into four groups with five mice each. Each group was orally treated with one compound at a dose of 250 mg/kg body weight while only the vehicle (90% PEG and 10% DMSO) was administered to the control group. Mice were not fasted for 3–4 h prior to dosing but had access to water ad libitum. The compounds were administered by esophageal gavage. The animals were observed using close monitoring during the first 24 h and once a day during the following 2 weeks in order to determine general behavioral changes, signs of toxicity or mortality. Animal weights were monitored and documented as well. After 14 days, the animals were humanely sacrificed by CO_2_ inhalation and the vital organs were examined for macroscopic alterations. Vital organs (liver, kidney, spleen, heart and lungs) were excised, weighed and stored in 10% formalin for later histological analysis.

### Evaluation of in vivo anthelmintic activity

#### *Collection of Haemonchus contortus third stage larvae (L*_*3*_*)*

One Merino sheep (12 weeks of age) was used and kept under controlled housing and feeding conditions at the facilities of the Instituto de Ganadería de Montaña (CSIC, León, Spain) to provide *H. contortus* L_3_. Before the experimental infection, the lamb was dewormed with ivermectin at 0.25 mg/kg body weight. The absence of trichostrongylid eggs in the faeces was confirmed after measuring the faecal egg count (FEC) by the McMaster technique. The sheep was orally infected with 250 infective larvae (L_3_) of *H. contortus* per kilogram of body weight and the infection was confirmed 21 days later by McMaster technique. Then, the faeces were pooled in plastic containers and cultured under moist aerated conditions at 25 °C for 7 days to allow the development of hatching eggs to L_3_^38^.

#### In vivo* bioassay on Mongolian gerbils*

This assay is based on previous a protocol^25–27^. Briefly, 36 female Mongolian gerbils (*Meriones unguiculatus*) weighting 40 g average, were randomly housed in polypropylene cages containing wood shavings for bedding at the facilities of the animal house of the University León (Spain). Commercial rodent chow and tap water were given ad libitum. Following a 7 days’ acclimation period, gerbils were immunosuppressed by adding hydrocortisone to the drinking water up to a final concentration of 1 mg/L for the duration of the study to allow better establishment of infection. After the animals had received medicated water for 5 days, 1 month old *H. contortus* infective larvae were exsheathed using carbon dioxide as previously described by Conder and Johnson^39^. Gerbils were orally dosed with 1000 *H. contortus* L_3_ in Earle's Balanced Salt Solution using an 18-gauge dosing gavage fitted to a 1-mL syringe. On day 10 post infection, animals were assigned randomly in groups of six animals per cage: group A (AAD-1 at 200 mg/kg), group B (AAD-1 at 10 mg/kg), group C (AAD-2 at 200 mg/kg) group D (2aBZ at 200 mg/kg), group E (2aBZ at 10 mg/kg) and group F (negative control). AAD-14 was not tested at a dose of 10 mg/kg due to compound shortage. Each compound was dissolved in an appropriate vehicle (90% PEG300 and 10% DMSO) and the desired dose was administered orally to each gerbil in a total volume of 0.4 mL via 18-gauge dosing gavage fitted to a 1-mL syringe. Control animals were dosed in an identical manner with the vehicle alone. On day 13 post infection (day 3 after treatment), all gerbils were humanely sacrificed by CO_2_ inhalation, their stomachs were removed, longitudinally opened, and put in glass vials containing 14 mL distilled water. The vials were vortexed and placed in a water bath at 37 °C for 5 h to allow the migration of the larvae to the liquid. Following incubation, the stomachs were removed and 1 mL of 37% formaldehyde solution (Sigma-Aldrich, Spain) was added to each vial for subsequent examination of L_4_ and L_5_ or pre-adults worms in five aliquots using a dissecting microscope (15–45x).

#### In vivo* bioassay on sheep*

This experiment was performed according to the recommendations of the World Association for the Advancement of Veterinary Parasitology (WAAVP) guidelines^40^. A total of eight male Merino breed lambs of 4 months years old were used and housed at the facilities of the “Instituto de Ganadería de Montaña” (CSIC, León, Spain). At the beginning of the experiment, animals were treated with ivermectin at 0.25 mg/kg live body weight to remove any burden of parasites and the faecal egg count (FEC) was performed 2 weeks later to confirm the absence of infection by a modified McMaster technique by counting all the eggs in each chamber (total volume 1 ml and multiply by 15 to give the eggs per gram or epg) and using a saturated sodium chloride (density = 1.2 g/ml). All animals showed an egg count of 0. The sensitivity of this technique is 15 epg. After a 3 weeks acclimatization period, animals were infected with a total of 250 infective third-stage larvae (L_3_) of *H. contortus* per kg body weight. FEC was performed on day 21 post infection to confirm the establishment of infection and on day 28 animals were randomly allocated in two different groups based on weight and parasite burden and orally treated: Group 1 (treated with 2aBZ at 120 mg/kg body weight) and group 2 or control group (treated with the vehicle, 90% PEG300 and 10% DMSO). One week later, on day 35 post infection, all animals were humanely slaughtered. At the necropsy, the abomasa were removed and the number of adult worms present in the lumen and mucosa were counted. For this, each abomasum was opened, thoroughly washed and the content was placed in a container for further examination. As the expected number of worms present in the control group was higher than 500, a sample of 5% of the total volume from the content of the abomasum was examined. In the case of the treated group, according to the FEC performed previously, less than 100 worms were expected to be present, so the total content was counted.

In addition, a faecal egg count reduction test (FECRT) was carried out to determine the percentage reduction of eggs eliminated by the animals from the treatment day to the slaughter day (day 7 treatment). Faecal samples were analyzed individually by a modified McMaster technique^40^. The FECR was calculated according to Coles et al.^41^:

FECR % = (1 − T/C) × 100, where T and C are the means of FEC in the treated and control groups, respectively.

### Evaluation of in vivo toxicity on gerbils and sheep

In order to assess the toxicity of the compounds on gerbils and sheep, animals were observed daily for any abnormal clinical signs or mortality and weighted. The animal's vital organs were visually inspected during the necropsy to discard possible macroscopic lesions produced by the compounds. Heart, liver, lungs, kidney and spleen were macroscopically analyzed and removed to carry out the histopathological analysis in both gerbils and sheep. In the case of sheep also brain, small intestine and abomasum samples were collected. All samples were fixed and preserved in 10% formalin. Tissues were trimmed and conventionally processed for embedding in paraffin wax. Sections of 4 µm thick were stained with hematoxylin and eosin and observed under a light microscope.

The histopathological study was only carried out in those gerbils treated with 2aBZ at a dose of 200 mg/kg and in the control group, after the results obtained in the in vivo efficacy assay performed with sheep.

### Data analysis

All experimental results were presented as mean ± SEM. The total number of worms recovered (L_4_, pre-adults or adults) were calculated for each group of animals. Efficacy of compounds in gerbils and sheep was calculated as percentage of parasite reduction: % reduction = 100 × (C − T/C), where C is the arithmetic mean number of worms in the untreated control group and T is the arithmetic mean number of worms in each treatment group. Shapiro–Wilk test was performed to determine the normality of the samples in each experiment in order to determine between parametric and non-parametric tests. In the case of the in vivo efficacy experiment carried out with gerbils, the statistical significance between groups was analyzed using the non-parametric test Kruskal Wallis H test for number of L_4_ and pre-adults. Regarding to the in vivo efficacy experiment performed with sheep, the group comparison was carried out by using the non-parametric Mann–Whitney U test. Regarding to the in vivo experiments carried out with mice, the data were normally distributed so the statistical significance between groups was analyzed by means of one-way ANOVA followed by Tukey’s multiple comparison test. All statistical analysis was performed on SPSS Version 25.

### Ethical statement

The authors declare that the study is reported in accordance with ARRIVE guidelines. The handling and care of the animals were conducted in compliance with Spanish Act (RD 53/2013) and European Union Legislation (2010/63/UE). The protocols were approved by the Animal Care Committee of the Universidad de León (ULE, León, Spain).

## Supplementary Information

Supplementary Information.

## Data Availability

The datasets supporting the conclusions of this article are included within the article text and additional files.
