# Supplementary material for: Novel compound shows in vivo anthelmintic activity in gerbils and sheep infected by Haemonchus contortus
Source: Sci Rep. 2022 Jul 29;12:13004. doi: 10.1038/s41598-022-17112-3 (PMC9338094; doi:10.1038/s41598-022-17112-3)
Supplement: Supplementary file 1 — Supplementary Information. [file 41598_2022_17112_MOESM1_ESM.docx]

| **GROUPS** | **Dose** | **1^st^ day Body Weight (g)** | **14^th^ day Body Weight (g)** |
| --- | --- | --- | --- |
| **2aBZ** | 250 mg/kg | 23.4 ± 0.21 | 25.9 ± 0.86 |
| **AAD-2** | 250 mg/kg | 23.5 ± 0.43 | 26.8 ± 0.61 |
| **AAD-1** | 250 mg/kg | 22.8 ± 0.39 | 26.8 ± 0.61 |
| **Control (vehicle)** |  | 22.0 ± 0.60 | 25.6 ± 0.71 |

**Table S1.** Effects of single oral dose administration 2aBZ, AAD-1 and AAD-2 on body weight of mice in acute toxicity study. Values are presented as mean ± SEM (n=5).

| **Organs** | **Control** | **2aBZ** | **AAD-2** | **AAD-1** |
| --- | --- | --- | --- | --- |
|  |  |  |  |  |
| **Kidney** | 0.83 ± 0.02 | 0.78 ± 0.02 | 0.73 ± 0.06 | 0.80 ± 0.06 |
| **Heart** | 0.77 ± 0.10 | 0.69 ± 0.05 | 0.64 ± 0.04 | 0.63 ± 0.05 |
| **Liver** | 6.35 ± 0.56 | 6.41 ± 0.34 | 6.32 ± 0.61 | 6.21 ± 0.22 |
| **Spleen** | 0.71 ± 0.07 | 0.55 ± 0.06 | 0.66 ± 0.07 | 0.57 ± 0.09 |

**Table S2.** Effects on organ to body weight indices in mice treated with a single dose of 2aBZ, AAD-1, AAD-2 and vehicle control group. Values are presented as mean ± SEM (n=5). Organ to body weight index = (organ weight / body weight) x 100.

| **Groups** | **Dose** | | **1^st^ day body weight (g)** | **10^th^ day body weight (g)** | | **13^th^ day body weight (g)** | |
| --- | --- | --- | --- | --- | --- | --- | --- |
| **2aBZ** | 200 mg/kg | | 46.80 ± 1.09 | 47.95 ± 1.06 | | | 47.17 ± 1.45 |
| **2aBZ** | 10 mg/kg | | 43.10 ± 1.44 | 45.85 ± 2.08 | | | 44.83 ± 2.24 |
| **AAD-2** | 200 mg/kg | | 46.72 ± 0.94 | 47.75 ±1.44 | | | 48.00 ± 1.13 |
| **AAD-2** | 10 mg/kg | | 46.73 ±0.88 | | 49.05 ± 1.68 | | 49.33 ± 1.78 |
| **AAD-1** | 200 mg/kg | | 46.28 ± 1.18 | | 48.50 ± 1.49 | | 48.50 ± 1.57 |
| **Control (vehicle)** |  | | 44.53 ± 1.60 | | 45.18 ± 1.62 | | 45.33 ± 2.17 |
|  | |  | | | | | |

**Table S3.** Tolerance of 2aBZ, AAD-2 and AAD-1 on gerbils infected with *H. contortus*. Body weights were registered at 1, 10 and 13 days after administration. Values are presented as mean ± SEM; N=6

| **Animal** | **Group of treatment** | | **EPG day 0 treatment** | | **EPG day 7^th^ treatment** | |
| --- | --- | --- | --- | --- | --- | --- |
|  |  |  |  |  |  |  |
| **A** | Non-treated | | 5,730 | | 8,270 | |
| **B** | Non-treated | | 4,710 | | 7,020 | |
| **C** | Non-treated | | 3,930 | | 1,950 | |
| **D** | Non-treated | | 2,895 | | 9,630 | |
| **E** | Treatedwith 2aBZ | | 5,550 | | 15 | |
| **F** | Treatedwith 2aBZ | | 4,965 | | 0 | |
| **G** | Treatedwith 2aBZ | | 3,525 | | 0 | |
| **H** | Treatedwith 2aBZ | | 3,195 | | 195 | |
|  | |  | |  | |  |

**Table S4.** Faecal egg counts of each animal before and after treatment with 2aBZ at a dose of 120 mg/kg. EPG: egg per gram of faeces.
